# Method for Optimal Sensor Deployment on 3D Terrains Utilizing a Steady State Genetic Algorithm with a Guided Walk Mutation Operator Based on the Wavelet Transform

**DOI:** 10.3390/s120405116

**Published:** 2012-04-19

**Authors:** Numan Unaldi, Samil Temel, Vijayan K. Asari

**Affiliations:** 1 Turkish Air Force Academy, Istanbul 34149, Turkey; E-Mail: s.temel@hho.edu.tr; 2 Department of Electrical and Computer Engineering, University of Dayton, Dayton, OH 45469, USA; E-Mail: vasari1@udayton.edu

**Keywords:** sensor deployment, 3D terrain, wavelet transform, genetic algorithm

## Abstract

One of the most critical issues of Wireless Sensor Networks (WSNs) is the deployment of a limited number of sensors in order to achieve maximum coverage on a terrain. The optimal sensor deployment which enables one to minimize the consumed energy, communication time and manpower for the maintenance of the network has attracted interest with the increased number of studies conducted on the subject in the last decade. Most of the studies in the literature today are proposed for two dimensional (2D) surfaces; however, real world sensor deployments often arise on three dimensional (3D) environments. In this paper, a guided wavelet transform (WT) based deployment strategy (WTDS) for 3D terrains, in which the sensor movements are carried out within the mutation phase of the genetic algorithms (GAs) is proposed. The proposed algorithm aims to maximize the Quality of Coverage (QoC) of a WSN via deploying a limited number of sensors on a 3D surface by utilizing a probabilistic sensing model and the Bresenham's line of sight (LOS) algorithm. In addition, the method followed in this paper is novel to the literature and the performance of the proposed algorithm is compared with the Delaunay Triangulation (DT) method as well as a standard genetic algorithm based method and the results reveal that the proposed method is a more powerful and more successful method for sensor deployment on 3D terrains.

## 1. Introduction

Optimal sensor deployment on 3D terrains is the problem of placing the sensors at the most appropriate spots in order to maximize coverage of a wireless sensor network (WSN). WSNs have a key role in today's data acquisition networks where the sensors of a WSN are deployed on environments for detection and surveillance purposes. The sensors can vary from fire detectors, seismographs, cameras to vital sign sensors on soldiers in a battlefield and data gathered from the sensors is usually sank into a base station which is connected to a network backbone [1]. However, there are many environmental challenges which affect the performance of a WSN; that is, the limited number of sensors, and dependency in the determination of the best location of each sensor on the terrain and sensor characteristics. In order to obtain the most efficient quality of coverage (QoC) measure on a terrain, robust deployment strategies have to be taken into consideration, in the sense that, optimal sensor emplacement enables us to minimize the manpower, time and the number of sensors. Sensors are essentially battery operated and consume energy during the transmission, reception and sensing phases. In order to maximize the network lifetime, reliable methods for sensor deployment to reduce the energy consumption in WSN is a vital issue.

Sensor deployment can either be stochastic or deterministic. In a stochastic deployment method, sensors are randomly deployed with a normal distribution scheme. However this is far from being effective because random deployment may cause sensors to be centralized or to be blocked by terrain features causing non line of sight (LOS) sensor spots. Hence this will ultimately decrease the probability of detection and sensing in the environment. With a deterministic method, sensors are deployed according to a predefined constraint such as; predetermined priority-regions on a field are equipped with more sensors in order to maximize the QoC. However, when the number of sensors is limited there will be coverage holes. Although both methods have their own advantages and disadvantages, they both fail to provide solutions to the problem of determination of the location coordinates of a predefined number of sensors which maximizes the coverage within a predefined 3D terrain. This is a kind of an NP-hard Minimum Set Cover (MSC) problem where the decision space grows exponentially with wider terrains. For example, within a map size of 1,024 × 1,024 pixels, there are 2^20^ possible sensor locations. With 128 sensors, there are [2^20^ (2^20^-1) (2^20^-2)….. (2^20^-127)] possible sensor deployment schemes. Thus, the huge decision space necessitates a heuristic search algorithm.

As a search algorithm, an elitist and a steady state genetic algorithm (GA) have been utilized to track the optimal placement schemes of sensors on a 3D region. The GA is an optimization technique which is based on an adaptive mechanism of biological systems [2]. Two widely used GA techniques are the Standard-GA (S-GA) [3] and the Steady State-GA (SS-GA) [4]. In S-GA, new offspring are born from the parents of an old population using the crossover and mutation operators (genetic operators) and these individuals become the new population. The new population gets old when the whole new population is created and the algorithm iterates until a termination condition is achieved [5,6]. The SS-GA is different from the S-GA that there is only one new child inserted into the new population at each generation. The performance of a GA is highly problem specific and depends on the utilized parameters. Therefore, modeling and determination of the parameters is crucial for finding an optimal solution for a problem. Hence, in this study various methods with a wide parameter range have been evaluated and we have come up to the solution that S-GA and SS-GA methods both give satisfactory results and SS-GA overwhelms the S-GA in terms of number of iterations.

In this paper, first two deployment strategies are investigated *i.e.*, the random deployment method and the Delaunay triangulation method [7]. With these two strategies, optimal solutions could not be achieved, thus a genetic algorithm based deployment strategy has been developed, in which each sensor is moved to a new position which bears an attractive force to change the current position of a sensor within the area of interest. The attractive force is estimated by taking the wavelet transformation of the coverage matrix of the area of interest (AoI) and the result is the pixel which has the minimum energy level and deserves to carry a sensor. To evaluate the attractive force and determine the energy bearings of pixels, WT of QoC matrix is used in an image segmentation sense. The main reason for choosing a wavelet transform approach for segmentation of the QoC matrix is that, it is able to analyze signals with non-stationary spectra and gives better and faster results than other transforms [8]; and to the best knowledge of the authors of this article, this is the first study which utilizes a WT based approach for deploying sensors on 3D terrains.

Moreover, most of the sensor deployment algorithms in the literature deal with two-dimensional (2D) zones and do not propose strategies to handle coverage in three-dimensional domains, which is more realistic and a requirement for both civilian and military applications. The deployment of sensors to achieve desired QoC levels is basically more challenging on 3D terrains compared to 2D terrains. In 3D environments, a LOS algorithm is needed in order to determine whether a point on the terrain is blocked by any obstacle or not, thus the complexity of the problem increases. In this paper, Bresenham's LOS algorithm has been employed owing to its faster computation, in the sense that it does not require interpolation calculations and requires less number of calculation points [9].

The paper is organized as follows: In Section 2, related work on sensor deployment methods which are developed for 3D terrains is reviewed. In Section 3, some preliminaries and problem model are presented and in Section 4, the proposed algorithm is explained and performance evaluations are presented. The paper is concluded in Section 5.

## 2. Related Work

The studies on sensor deployment, especially for 3D terrains, usually take into account that the number of the sensors is constant. With a given number of sensors, the goal is to achieve maximum sensor coverage, thus maximum network utilization, minimum energy consumption or both.

Wang *et al.* [10] propose a genetic algorithm-based sensor deployment method, which deals with the problem of maintaining sensing coverage by a small number of sensors and low energy consumption in a wireless sensor network consisting of directional sensors [10]. They consider the priority-based target coverage problem and try to find a minimum subset of directional sensors that can monitor all targets, satisfying their prescribed priorities. Jia *et al.* propose a coverage control scheme based on elitist non-dominated sorting genetic algorithm (NSGA-II) in which a small number of sensor nodes are kept active to decrease the energy consumption [11]. They consider a large number of sensors with adjustable sensing radius that are randomly deployed to monitor a target area. Bakhtari *et al.* presented an implementation of a surveillance system in which multiple active-vision sensors are utilized [12]. In their implementation the position and orientation of a single target are tracked along its unknown trajectory. An optimal subset of dynamic sensors which moves in response to the motion of the target, are used for data-fusion process. Tezcan and Wang propose a new algorithm which tackles the coverage and orientation problem for video sensor networks [13]. Their aim is to find the most beneficial orientations for all multimedia sensors to maximize multimedia coverage in the 2-D case for improving quality of the information sensed from the region of interest, simultaneously minimizing the negative effect of occlusions and overlapping regions in the sensing field. Mittal and Davis propose a method to maximize the visibility from static sensors in a dynamic scene in which moving objects may occlude each other [14]. They determine the optimum number and placement of cameras in the scene for various scenarios.

There are various deterministic sensor deployment examples in the literature. One of the most successful deployment methods is to place each sensor in the middle of a Delaunay triangulation or the middle of Voronoi polygons of sensor coordinates [7]. To deploy a minimum number of relay nodes, Senel and Younis utilize the Triangular Steiner Tree approximation [15]. Another localization method is proposed by Chen *et al.*, which first splits the target region into sub-grids [16]. By deploying sensor nodes which reside on the vertex of each grid, the blind nodes are determined by comparing their Received Signal Strength Indicator (RSSI) values in order to deploy minimum number of sensors. For the case of mitigating the coverage holes after an initial random deployment, Chizari *et al.* [17] propose a divide-and-conquer algorithm based on a Delaunay triangulation method and propose a new sensing coverage method, which provides more detailed QoC information than its predecessors about the uniformity of coverage, which has a remarkable influence on network efficiency. However, this method fails to prove whether the triangulation-based deployment leads to optimal solutions or not. Stochastic deployment approaches usually make assumptions on the probability distribution of the sensor deployment onto the terrain. Fekete *et al.* [18] propose a random distribution of sensors inside a geometric region according to the boundary detection algorithm. In their approach they suggest the idea that the boundary nodes would have lower degrees than that of the interior ones and provide a degree threshold to differentiate interior and boundary nodes. Also in [19], Wang and Zhong propose a polynomial-time approximation algorithm to find a solution to the problem of deploying minimum number of sensors on a bounded 3D field. A grid distribution and a greedy heuristic are introduced to determine the best placement of sensors.

Moreover, there are various heuristic deployment strategies in the literature. For example, in [20], the Artificial Bee Colony (ABC) algorithm is applied to the dynamic deployment of sensor nodes in order to increase the coverage area of the network. A Simulated Annealing (SA) method and a Tabu Search (TS) method are proposed in [21] and [22], respectively. In [23], Kulkarni and Venayagamoorthy propose a sensor deployment strategy based on bio-inspired algorithms, particle swarm optimization (PSO) and bacterial foraging algorithm (BFA) for image segmentation. Their study reveals that bio-inspired algorithms perform multilevel image segmentation faster than the exhaustive search for optimal thresholds. In another study, Topcuoglu *et al.* propose a method for deployment of sensors on a 3D terrain with a hybrid-evolutionary algorithm [24]. In this study, authors take into account multiple objectives, however their model is particularly based on sensors which require defining the conic field of (camera) view analysis of each deployed sensor. On the other hand, in the proposed work, the usage of generic omni-directional sensors is assumed, where the sensing region is specified as a cube. This puts our study to be a generic model for every type of sensors.

In this paper, in order to mitigate the coverage holes after the initial deployment of a number of sensors, a wavelet transformation based mutation operator is utilized, which effectively gives better coverage results. Also, as stated above, most of the studies on sensor deployment problem take into account 2D terrains which is not sufficiently accurate for outdoor applications whereas in this study different types of 3D terrains ranging from rough and undulating ones to smooth ones are considered. Also according to the literature mentioned above, we apply more real-world-like input factors such as sensor coverage capabilities, terrain and sensor features, *etc.*

## 3. Preliminaries

The primary objective of this study is to maximize the overall QoC of a WSN when deploying a specific number of sensors on a 3D surface. Some GA approaches are empirically tested for the search of an optimal deployment scheme. By starting with the same initial population the search performance of an S-GA and an SS-GA have been evaluated. In this section, firstly the problem model and preliminaries are given, secondly the results for two widely used deployment methods are shown and lastly the proposed deployment algorithm is presented.

In order to make fair comparisons and evaluate the performance of the deployment methods, the algorithms are run with the same parameters such as the interested terrain, the sensor types, coverage calculation algorithm, LOS algorithm, initial population *etc.* Afterwards, the convergence speed with regard to QoC rate is analyzed.

### 3.1. The Problem Model

**Figure 1.**
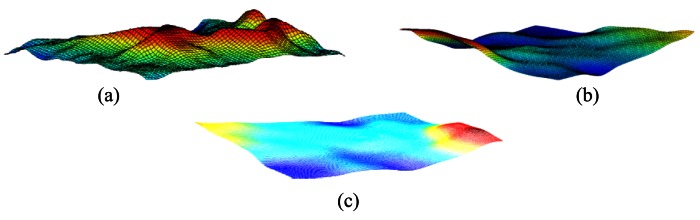
Examples of terrain types used in this study. (**a**) A rough terrain; (**b**) An undulating terrain; (**c**) Smooth terrain.

The sensor deployment is a challenging task, in the sense that different terrains (and also sub-regions of a terrain) may exhibit coverage holes after an initial deployment scheme. In this study, three different 3D terrain types are used, *i.e.*, rough terrains, undulating terrains to smooth terrains. Examples for the 3D terrains which are used in this study are shown in Figure 1.

**Figure 2.**
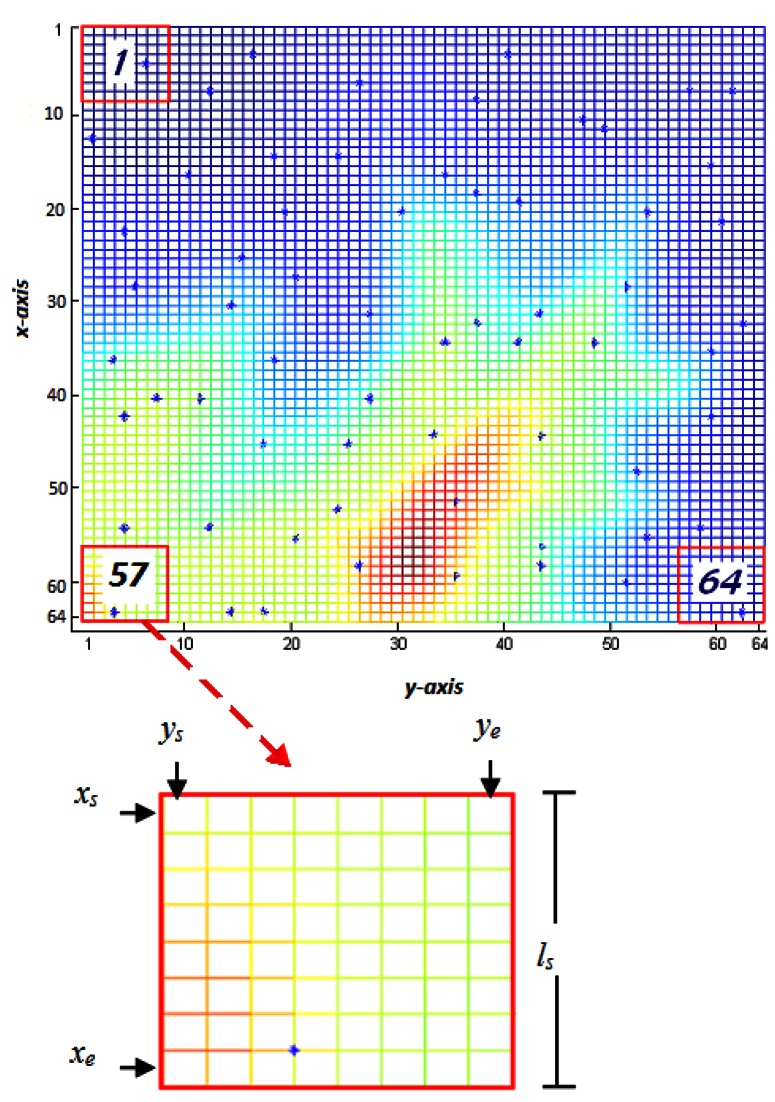
The terrain, sub-regions, and pixel coordinate notations.

The problem takes into account a terrain which is denoted as *T*, where *N* sensors will be deployed. The terrain has a size of *M*×*M* pixels. As an initial setup, every pixel in the terrain is numbered with Equation (1) and denoted as *P_n_* and when *P_n_* is given, the Cartesian coordinates (*x, y*) are calculated with Equation (2) and Equation (3) respectively:

(1) $$P_{n}=(y-1)\times M+x$$

(2) $$y=\left\lfloor \left(P_{n}-1\right)/M\right\rfloor +1$$

(3) $$x=P_{n}-M\times (y-1)$$

where ⌊·⌋ denotes the floor operator. The terrain *T* is divided into *N* sub-regions. The pixel length of each sub-region *l_s_* is calculated with the formula, $l_{s}=\left\lfloor M/\sqrt{N}\right\rfloor$ where *M* denotes the pixel length of each dimension of terrain *T*. *T_i_* denotes the sub-region number with *i*=*1, 2, …N*. The number of sub-regions is equal to the number of sensors and each sub-region is assigned with only one sensor for an initial deployment. The start and end coordinates of every sub-region in the *x* axis, *x_s_* and *x_e_*, are determined by Equation (4) where ⌈·⌉ is the ceiling operator. The start and end coordinates of every sub-region in the *y* axis, *y_s_* and *y_e_*, are determined by Equation (5) where *mod*() defines the modulo operator. As shown in Figure 2, a sub-region is bounded with coordinates from *x_s_* to *x_e_* in the vertical axis and from *y_s_* to *y_e_* in the horizontal axis (MATLAB^®^ notation). As it can be seen in Figure 2, for the region which has the sub-region number, *T_i_* = 57 and sub-region length, *l_s_* = 8; the starting and ending *x*-axis coordinates will be *x_s_* = 57 and *x_e_* = 64, and the starting and ending *y*-axis coordinates will be *y_s_* = 1 and *y_e_* = 8 respectively:

(4) $$\begin{array}{l} x_{e}=\left\lceil T_{i}/l_{s}\right\rceil \times l_{s}\\ x_{s}=x_{e}-l_{s}+1 \end{array}$$

(5) $$\begin{array}{c} y_{e}=(\text{mod}\left(T_{i}-1,l_{s}\right)+1)\times l_{s}\\ y_{s}=y_{e}-l_{s}+1 \end{array}$$

### 3.2. The Sensing Model

**Figure 3.**
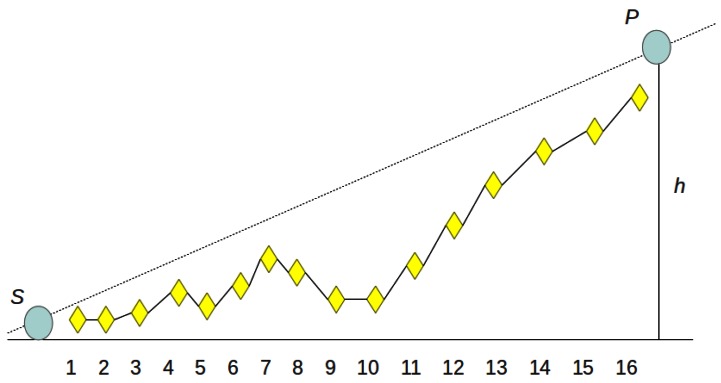
Determination of LOS between a sensor and a phenomena.

The sensing model utilized in this study is a probabilistic model which allows a realistic modeling of sensor coverage probability [7]. In this model the sensed phenomenon is defined at location *p* for the sensor *s* with a predefined sensing range as *s_r_* and an uncertainty sensor detection range defined as *u_r_* where *u_r_* <*s_r_*. If the sensed phenomenon *p* lies before the range (*s_r_*–*u_r_*) and sensor *s* is not occluded by any obstacle (there is LOS between *s* and *p*), then it is certainly sensed. When *p* lies within (*s_r_*–*u_r_*) and (*s_r_* + *u_r_*) and if there is LOS between *s* and *p*, then the detection probability can be expressed as *exp(-α.dist^β^)*. When *p* lies out of the range (*s_r_* + *u_r_*) or if there is a non-line of sight (NLOS) then it is certainly not sensed. In this paper, we have utilized the well known Bresenham LOS algorithm for LOS detection. Owing to its integer computation, it yields faster computation, does not require interpolation calculations and requires a lesser number of calculation points [9]. Although Bresenham's algorithm is generally used in computer graphics for line drawing on 2D surfaces, we have modified it to be used for LOS determination on 3D spaces. Figure 3 shows a simple LOS scenario. As shown in the figure, the height of any corresponding pixels does not cut the virtual line drawn from a sensor *s* and a phenomenon *p*, hence there is a LOS between *s* and *p*.

The sensing probability *O_q_(s,p)* of the probabilistic sensing model with Δ *(s,p)* denoting the 3D Euclidian distance between *p* and *s* can be expressed as follows:

(6) $$O_{q}\left(s,p\right)=\{\begin{array}{cc} 1, & \Delta \left(s,p\right)\leq \left(s_{r}-u_{r}\right)\;\text{and if LOS}\\ e^{-\alpha .\text{dis}t^{\beta }}, & (s_{r}-u_{r})<\Delta \left(s,p\right)\leq (s_{r}+u_{r})\;\text{and if LOS}\\ 0, & \Delta \left(s,p\right)>\left(s_{r}+u_{r}\right)\;\text{or if NLOS} \end{array}$$

(7) $$\text{dist}=\left(\Delta \left(s,p\right)-\left(s_{r}-u_{r}\right)\right)/2\times u_{r}$$

The overall map of sensing probabilities of each location constitutes the so-called QoC matrix. The values of *α* and *β* reflects the environmental characteristics of the terrain. By carefully adjusting these variables, various sensor and terrain types can be defined.

### 3.3. The Discrete Wavelet Transform

Wavelets, which are used for representing data or other functions, group data into various frequency components to work on each component separately at each scale. Compared to traditional transformation methods, wavelet analysis has advantages in analyzing physical situations, especially when the signal contains discontinuities and sharp spikes [25]. Wavelets are utilized in the fields of applied mathematics, electrical engineering, image processing, *etc.*

Two-dimensional implementation of the discrete wavelet transform (DWT) is commonly used in image-processing applications. The DWT provide spatial (or temporal) and frequency information (*i.e.*, space-frequency or time-frequency analysis) simultaneously and is widely used in the analysis of transientor time varying signals. WT approach is able to analyze signals with non-stationary spectra and gives better and thus faster results than other transformations.

**Figure 4.**
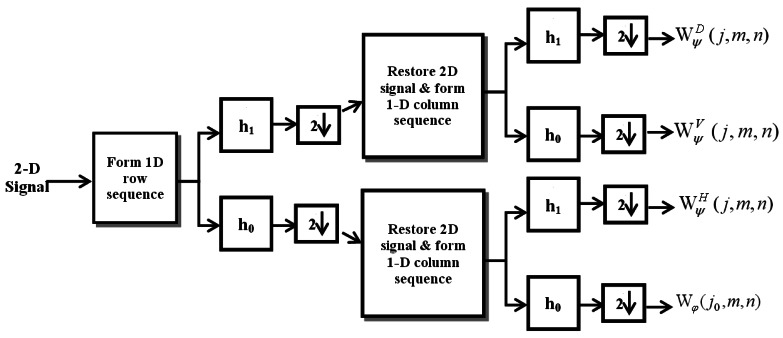
The analysis filterbank for 2D fast wavelet transform for one-level decomposition.

Any two dimensional signal *f(x, y)* of size M × N is decomposed by using 2D discrete wavelet transform is given in Equation (8):

(8) $$\begin{array}{c} {\text{W}}_{\varphi }(j_{0},m,n)=\frac{1}{\sqrt{MN}}\sum_{x=0}^{M-1}\sum_{y=0}^{N-1}f(x,y)\varphi_{j_{0},m,n}(x,y)\\ {{\text{W}}^{i}}_{\psi }(j,m,n)=\frac{1}{\sqrt{MN}}\sum_{x=0}^{M-1}\sum_{y=0}^{N-1}f(x,y){\psi^{i}}_{j,m,n}(x,y)\;i=\{H,V,D\} \end{array}$$

where W*_φ_*(*j*_0_, *m, n*) are the approximation (scaling) coefficients at level *j_0_* and *φ*_*j*0,*m*,*n*_ are the scaling functions; ${\text{W}}_{\psi }^{i}(j,m,n)$ are the detail (wavelet) coefficients at scales *j* ≥ *j*_0_ and *ψ^i^_j,m,n_*(*x, y*)are the corresponding wavelet functions with *i* = {*H,V,D*} representing horizontally, vertically and diagonally sensitive wavelets. While W*_φ_*(*j*_0_,*m,n*) represents an approximation to *f(x,y)* and embodies the energy compaction of the given signal, W*^i^_ψ_*(*j,m,n*) represent the highpass or the detail components which characterizes the signal's high frequency information with horizontal, vertical and diagonal directions, respectively [25]. At each level of the standard DWT, the size of approximate coefficients and detail coefficients decreases by a factor of 2 resulting in a perfectly non-redundant of O(n) representation of a given signal. The sparse representation with energy compaction makes the standard DWT widely accepted for signal compression. In our implementation, the 2D fast DWT is employed using 1D digital filters based on the separable 2D scaling and wavelet functions and downsamplers. The scheme for taking the fast DWT is depicted in Figure 4. One-level decomposition filterbank shown in the figure can be iterated on W*_φ_*(*j*_0_,*m,n*) by binding it to the input of another filterbank to provide multilevel decomposition.

In this study, the overall QoC is regarded as a measure of signal level provided by the neighboring sensors in each location (pixel). In order to determine the coverage holes in a sub-region we take the DWT of the sub-QoC matrix. The minimum value in the resulting approximation matrix of the wavelet transform gives the least energy bearing pixel point which corresponds to an area in the sub-region. This fact is used to relocate the sensor by moving it towards the least covered region.

### 3.4. Random and DT based Sensor Deployment

In random deployment, N sensors are scattered onto random coordinates of the terrain and the correspondent QoC is evaluated with the probabilistic sensing model. At each iteration, the coverage value and the sensor positions are recorded. This scheme is repeated several times and the QoC value is determined by taking the average. Although a Gaussian distribution can enable equally distributed sensors in a region, random deployment is far from being an optimal solution.

**Figure 5.**
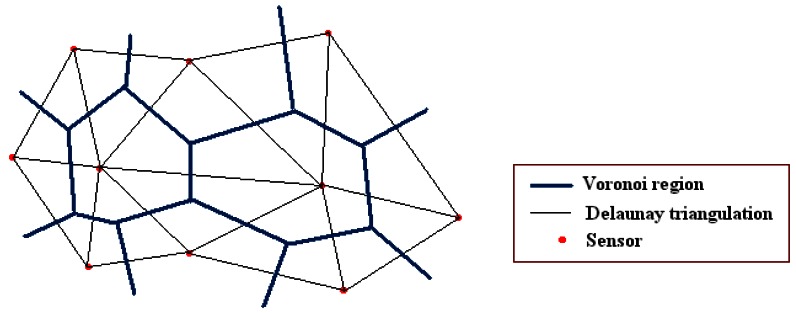
Voronoi regions and corresponding Delaunay triangulation.

The second deployment method which is evaluated in this paper is the Delaunay Triangulation (DT) based approach. The DT-Score algorithm proposed in [7] is adapted to be used on 3D terrain height-maps. DT is the dual graph of Voronoi diagram and an example of a Voronoi Diagram and its corresponding DT is given in Figure 5. The DT based deployment approach utilizes the idea of placing the next sensors to the uncovered regions. Before deploying a sensor, each candidate position is generated from the current sensor positions and a new sensor is placed into the middle of the largest empty circle in a DT. The algorithm is repeated until all the sensors are placed. One disadvantage of the algorithm is that the DT always yields to non-covered regions on the boundaries of the terrain. In order to overcome this disadvantage, at the very beginning of the deployment, sensors are manually deployed on the edges of the terrain. This ensures to accumulate the probability of the coverage of all the pixels in the region.

**Figure 6.**
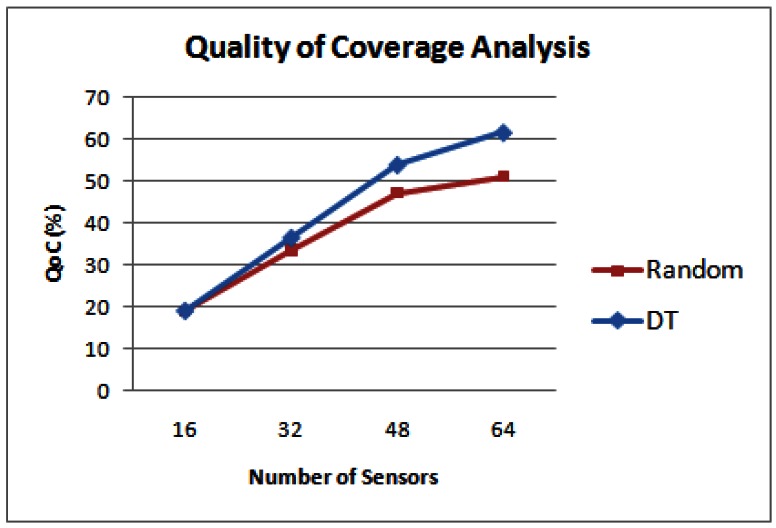
QoC results for random deployment strategy and Delaunay triangulation strategy.

In Figure 6, the performance evaluation results of the random deployment and the DT based method are shown. It can be inferred from Figure 6 that the DT based deployment approach is much more effective than a random deployment method in terms of QoC. However, DT based approach does not exhibit an optimal search strategy. This is evident from the coverage results achieved by applying the GA based deployment strategies for the same terrain, which are presented in Section 5.

## 4. The Proposed Methods

The GA is proven to be a robust and optimal search technique for various applications since it was first proposed by Holland [2]. It is based on the adaptive mechanism of biological systems. The structure of a GA is simple and straightforward: it iterates through fitness assessment, selection and breeding, and population reconstruction. The primary difference between the versions of GAs is in how the parents of a population are selected and how the breeding takes place. To breed, two parents are selected from the original population. The features of the parents are copied and recombined (crossovered) with each other and the results are mutated to form two children. This process is repeated until the child population is fulfilled. In order to avoid premature convergence, mutation operators are used to escape from local optima. The original technique for GA selection was called roulette wheel selection. In this selection method, individuals are selected in proportion to their fitness. Hence, if an individual has a higher fitness, its probability to be selected is higher. Elitism is another simple and yet successful concept in GAs. By deploying an elitist approach, the fittest individual or individuals from the previous population, which are called elites, are directly injected to the next population. This algorithm also has similar exploitation properties.

An alternative to a traditional S-GA approach is to use a SS-GA approach, where there is only one new (the fittest) child inserted into the new population at any generation. The idea is to iteratively produce a new child (or two), calculate their fitness, and then reintroduce them directly into the population itself, killing off some preexisting individuals to make room for them. The SS-GA has two important features. First, it uses less memory than S-GA because there is only one population at a time. Second, it is fairly exploitative compared to a traditional approach: the parents stay around in the population, potentially for a very long time. This problem can also be tackled with applying an appropriate mutation scheme.

### 4.1. Representation of Individuals

**Figure 7.**
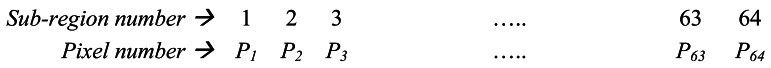
Individual representation of sensor deployment.

In this study, it is shown that S-GA and SS-GA methods produce better and more satisfying sensor deployment schemes. For both methods, integer representations of sensor pixel positions are used. As shown in Figure 7, one individual gene of a population represents one deployment scheme. As an example, with given 64 sensors, the terrain is divided into 64 equal sub-regions. For the terrain shown in Figure 2, sensors are deployed at 52nd pixel of sub-region-1, 5th pixel of sub-region-2, …, 48th pixel of sub-region-64. By applying Equations (1–5), it is also possible to convert the pixel numbers to Cartesian coordinates.

### 4.2. Fitness Function

The fitness function evaluates how well each parent (deployment of N sensors) covers the terrain. In other words, while each sensor has predefined coverage parameters, each sensor has an amount of coverage within a circle in its periphery. As stated in Section 3.1, if the sensed pixel p, is within the sensing range, *s_r_* and if there is LOS between two pixels, it is certainly sensed and the pixel *p* that corresponds to the coverage matrix is set to 1 because it is sensed with a probability of 100%. If the sensed phenomenon *p* lies within (*s_r_* − *u_r_*) and (*s_r_* + *u_r_*) then the sensing probability can be expressed as *exp(-α.dist^β^)*. If the distance between s and p lies far beyond (*s_r_* + *u_r_*), the sensor cannot sense the phenomenon on pixel p. The fitness function can be expressed as follows:

(9) $$F(i)=\frac{1}{P}\sum_{j=1}^{N}\left(\sum_{k=1}^{P}O_{q}\left(s_{j},p_{k}\right)\right)$$

where *F(i)* denotes the fitness value of the parent *i*; and *j* denotes the sensor number (or the sensor in sub-region *j*), *P* is the number of pixels in the whole map and *O_q_(s,p)* denotes the sensing probability which is bound to the distance between *s* and *p*. Since sensor *j* has coverage on a few pixels in its periphery, only those pixels within the coverage range contribute to the inner summation of Equation (9). In our implementation if the same pixel is covered by more than one sensor, only the one with the highest sensing probability is used in the calculation of *F(i)*. Ultimately the fitness value represents the percentage of the coverage amount (which we named as Quality of Coverage-QoC).

### 4.3. Recombination

In this study we have utilized the *single point crossover* technique because of its simplicity and ease of use. It is straightforward, in the sense that it does not expose any calculation burden on the algorithm. In addition, as stated in Section 4, the success of the algorithm depends highly on the mutation operator instead of recombination.

In this method, a random crossover index *P_c_* between [1, N] is determined, which represents the point of the crossover between two parents and after recombination two new children are born. In addition, 20 individuals survive in the population at each generation. In S-GA 20 new children are born whereas in SS-GA only one child is determined to be injected into the population. This population size is determined to be the best empirically. With smaller population sizes, the GA converges prematurely and with larger sizes the computational time grows exponentially.

### 4.4. Mutation

The mutation operator in our study is based on moving a sensor to a new pixel position within its sub-region. Two deployment strategies arise in our study: a simple deployment strategy (SDS) (random walk mutation) and a WT based deployment strategy (WTDS) (guided walk mutation). The SDS is straightforward that if the sensor is to be mutated, it is put to a randomly new pixel position that is within its periphery. The decision that a sensor will be mutated is given with a comparison of a constant *P_m_*, which denotes the probability of mutation. When the program iterates, a random number is generated. If this random number is less than or equal to *P_m_*, the gene (sensor location) will be mutated. *P_m_* is determined empirically and when it is selected to be 0.1, the algorithm performs the best.

**Figure 8.**
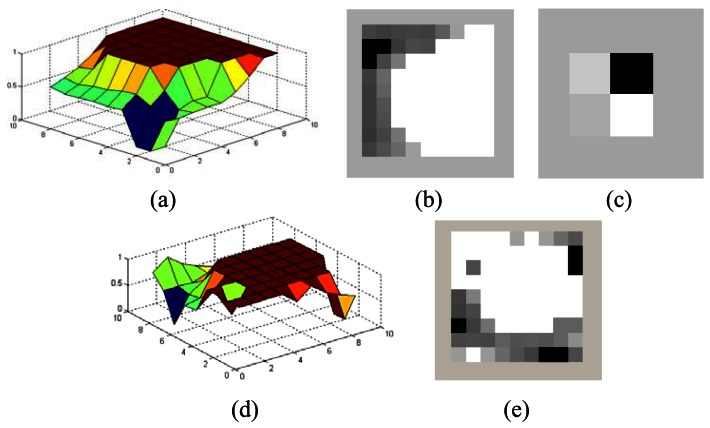
Process of WTDS with guided walk mutation (**a**) The QoC matrix of the sub-region before mutation; (**b**) The grayscale representation of the QoC matrix; (**c**) The approximation matrix after WT; (**d**) The QoC matrix of the sub-region after mutation; (**e**) The grayscale representation of the QoC matrix after mutation.

In WTDS, the next position of the sensor is determined with the help of WT. When a sensor is to be mutated, each sensor is moved to a new pixel position which bears an attractive force to change the current position of the sensor within the sub-region. The attractive force is estimated in two different ways, *i.e.*, by taking the wavelet transform of the coverage matrix of either the corresponding sub-region or of a region in the neighborhood of the sensor to be mutated. The resulting approximation matrix of the WT embodies compact energy (coverage) distribution in that region. The location of the pixel which has the minimum energy level in the approximation matrix corresponds to an area with the least sensor coverage in that region and that area enforces the sensor to change its location towards it. In the first approach, the new sensor stays in the same sub-region whereas in the second approach the new sensor location may be in another sub-region. This process is illustrated in Figure 8. In Figure 8(a) the QoC matrix for a region of 8 × 8 pixels is shown. According to this figure, the sensor sits on pixel coordinate (8, 7). In Figure 8(b), the gray-scale representation of the QoC matrix is given. The white pixels denote the fully covered pixels and the black pixels correspond to 0% coverage. Total QoC for this region is 71% on average. The WT of the QoC matrix for this region is taken with 2 levels. The resulting approximation matrix of the WT is shown in Figure 8(c) where one can see that the top-right pixel has the least energy level, that it attracts the sensor towards the corresponding area of size 4 × 4 pixels on that region. The sensor is then randomly moved to one of these pixel locations. In Figure 8(d,e), the resulting QoC matrix is shown where the QoC is increased to 74%.

### 4.5. Summary of the Parameters

In Table 1, the definitions and values of the parameters used for evaluating the proposed algorithm are listed.

## 5. Numerical Results

As stated above, in order to maximize the quality of coverage within a given terrain and a constant number of sensors, numerous versions of GAs are examined in order to find optimal sensor deployment schemes. We have come experimentally to the decision that S-GA and SS-GA methods with a population size of 20 raise more effective solutions than any other GA methods. Roulette wheel selection and an elitist approach are followed in order to sustain the best population of individuals.

Moreover, in order to evaluate the performance of the proposed mutation method, it is compared with a simple deployment strategy (SDS). In SDS, the mutation is realized by assigning a sensor to a new random pixel position within its periphery (1 to 5 pixels). On the other hand with the proposed WT based deployment strategy (WTDS), the sensor to be mutated is guided to a new pixel position which is determined by finding the least energy levels in its sub-region or in its surrounding region. Ultimately, the performances of six different methods which are listed in Table 2 are evaluated.

**Figure 9.**
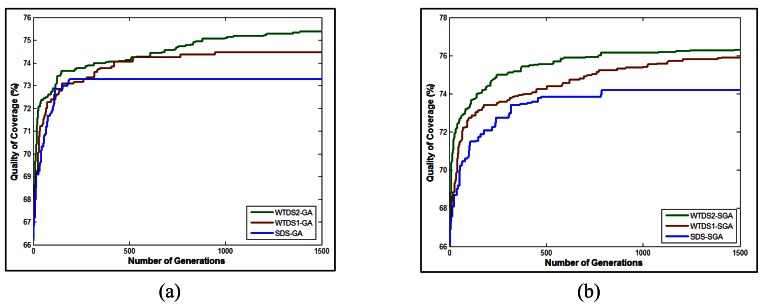
Comparison of the proposed methods (**a**) three standard GA methods (**b**) three steady state GA methods.

In Figure 9(a), the comparison of three standard GA approaches, SDS-GA,WTDS1-GA and WTDS2-GA is presented. As apparent from the figure, after 200 iterations, the SDS-GA approach prematurely converges. Also the maximum QoC rate after 1,500 iterations, achieved with SDS-GA is 73.3%. WTDS-GA methods give better QoC results than SDS-GA, that the maximum QoC rate that can be achieved with WTDS1-GA and WTDS2-GA are 74.5% and 75.4%, respectively. Since the mutations in WTDS-GA methods are guided, the search for the best sensor locations gives improved QoC results, albeit after about 500 iterations, WTDS1-GA approach also prematurely converges.

**Figure 10.**
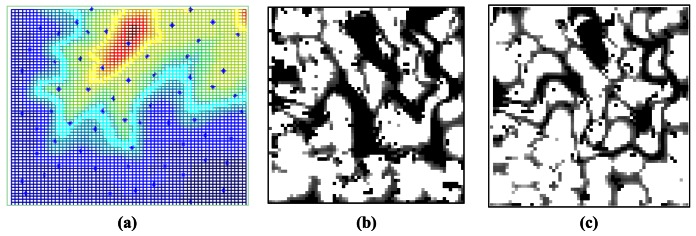
Final deployment results of WTDS-SGA method (**a**) optimally deployed sensors on the terrain; (**b**) the coverage matrix after first generation (black regions represent coverage holes); (**c**) the coverage matrix after last generation.

After detecting that a fully elitist GA approach results in convergence problems, a steady state approach is pursued both with SDS and WTDS based mutations where at each iteration, only the best child replaces the worst parent in the population. As shown in Figure 9(b), steady state method gives better results and the convergence is due after 1,500 iterations. With SDS-SGA method, the maximum QoC rate that can be achieved is 74.2%. WTDS1-SGA gives better QoC which is 75.9% and WTDS2-SGA gives better QoC results than any other deployment methods by rate of 76.3%. It is also noteworthy that a 2–3% increase in the QoC rate may seem insignificantly small. However, as stated in Equation (9), the QoC value represents an average amount, where any increase in this amount yields to a much better coverage. In addition, the final deployment of sensors can be seen in Figure 10(a). It can be inferred from the figure that with WTDS2-SGA, although each sub-region does not necessarily has a sensor inbetween its boundaries, the final positions of sensors are almost uniformly distributed around the terrain. It can be observed from Figure 10(b,c) that the proposed WTDS2-SGA method successfully reduces coverage holes on the terrain.

In our application, we have taken the sensor height from the ground, S*_h_* as zero, since we are dealing with sensors which are to be camuflated on the field for military purposes. Increasing the height of the deployed sensors to a reasonable height (e.g., 0.1*s_r_*) will increase the sensor coverage with ∼1.5% increase in QoC since some of the nonLOS phenomena locations with respect to a given sensor may become LOS due to raised sensor height. If the height of the sensor nodes are adjusted separately from eachother, it may be possible to get better QoC results. Nevertheless, as stated about the sensors are to be deployed just on the surface in the current work.

## 6. Discussion

As stated in Section 1 and Section 2, the optimal sensor deployment problem has NP-Hard complexity which necessitates heuristic approaches to be used. Although there are interesting and successful deployment methods, the Simulated Annealing [21] and Tabu Search [22] methods have relatively less time complexity and the ABC method [20] produce better results especially for dynamic environments. However, majority of them are proposed for 2D environments and lack LOS issues.

In this study we utilized different types of GAs and determined the coverage holes with WT which is a novel technique for this application. A drawback in our approach is that, the coverage and LOS calculations for 3D environments are more complex and computational time increases for larger terrains. Also our work aims to deploy stationary sensors. Evaluation of our method for dynamic environments and mobile sensors is an open issue. In addition, bio-inspired models have proven to be useful for solving deployment problems [26]. However, like many of the bio-inspired studies our study is also based on simulations. The performance of the algorithms have to be proven by empirical real-world scenarios.

## 7. Conclusions and Future Work

The deployment of limited number of sensors on 3D terrains to achieve maximum sensor coverage is a non-trivial task and studies of deploying in 2D environments are extensive, but methods for 3D environments are scarce. In the scope of this paper, we have focused on searching for optimal solutions with GAs. A wavelet transform-based guided walk mutation algorithm has been proposed in order to maximize the QoC levels. The performance results reveal that the proposed algorithms outperform the random deployment, the DT based deployment and the standard GA deploymentapproaches. Among the two approaches proposed for the mutation phase, the one which does not restrict more than one sensor deployment in a sub-region provides better QoC.

This study represents a novel and robust sensor deployment approach on 3D terrains for static sensors. Finding optimal solutions of sensor coverage for the sensors that can be placed at different heights on a mobile platform and equipped with communication facilities will be the next step.

## Figures and Tables

**Table 1.** Summary of parameters.

**Parameter**	**Description**	**Value**
*M*	Map length	64
*N*	Number of sensors	64
*s_r_*	Sensing range	6
*u_r_*	Uncertainty sensing range	1
*α and β*	Environmental characteristics	0.8 and 0.4
*P_c_*	Crossover point	14 to 54
*P_m_*	Mutation probability	0.1
*P_size_*	Population size	20
*S_h_*	Sensor Height	on Surface

**Table 2.** Evaluated GA methods.

**Algorithm**	**Description**	**Explanation**

SDS-GA	Simple sensor deployment strategy with a standard genetic algorithm approach	20 children born in each generation. 20 best are selected for the next population. Mutations are totally random.

WTDS1-GA	Wavelet transformation based sensor deployment strategy with a standard genetic algorithm approach	20 children born in each generation. 20 best are selected for the next population. Mutations are WT guided. The location of a mutated sensor is in its current sub-region.

WTDS2-GA	Wavelet transformation based sensor deployment strategy with a standard genetic algorithm approach	20 children born in each generation. 20 best are selected for the next population. Mutations are WT guided. The new sensor location may move to another sub-region after the mutation.

SDS-SGA	Simple sensor deployment strategy with a steady state genetic algorithm approach	20 children born in each generation. Best child replaces worst parent. Mutations are totally random.

WTDS1-SGA	Wavelet transformation based sensor deployment strategy with a steady state genetic algorithm approach	20 children born in each generation. Best child replaces worst parent. Mutations are WT guided. The location of a mutated sensor is in its current sub-region.

WTDS2-SGA	Wavelet transformation based sensor deployment strategy with a steady state genetic algorithm approach	20 children born in each generation. Best child replaces worst parent. Mutations are WT guided. The new sensor location may move to another sub-region after the mutation.
